# Selection of EMG Sensors Based on Motion Coordinated Analysis

**DOI:** 10.3390/s21041147

**Published:** 2021-02-06

**Authors:** Lingling Chen, Xiaotian Liu, Bokai Xuan, Jie Zhang, Zuojun Liu, Yan Zhang

**Affiliations:** 1 School of Artificial Intelligence and Data Science, Hebei University of Technology, Tianjin 300131, China; chenling@hebut.edu.cn (L.C.); 201832502013@stu.hebut.edu.cn (X.L.); liuzuojun@hebut.edu.cn (Z.L.); zy_ljh9@163.com (Y.Z.); 2 Engineering Research Center of Intelligent Rehabilitation Device and Detection Technology, Ministry of Education, Tianjin 300131, China; 3 School of Chemical Engineering and Advanced Materials, Newcastle University, Newcastle upon Tyne NE1 7RU, UK; jie.zhang@newcastle.au.uk

**Keywords:** EMG sensors, residual limb, functional network, convergent cross-mapping, directed network

## Abstract

The intelligent prosthesis driven by electromyography (EMG) signal provides a solution for the movement of the disabled. The proper position of EMG sensors can improve the prosthesis’s motion recognition ability. To exert the amputee’s action-oriented ability and the prosthesis’ control ability, the EMG spatial distribution and internal connection of the prosthetic wearer is analyzed in three kinds of movement conditions: appropriate angle, excessive angle, and angle too small. Firstly, the correlation characteristics between the EMG channels are analyzed by mutual information to construct a muscle functional network. Secondly, the network’s features of different movement conditions are analyzed by calculating the characteristic of nodes and evaluating the importance of nodes. Finally, the convergent cross-mapping method is applied to construct a directed network, and the critical muscle groups which can reflect the user’s movement intention are determined. Experiment shows that this method can accurately determine the EMG location and simplify the distribution of EMG sensors inside the prosthetic socket. The network characteristics of key muscle groups can distinguish different movements effectively and provide a new strategy for decoding the relationship between limb nerve control and body movement.

## 1. Introduction

The number of disabled persons is increasing every year due to accidents such as work-related injuries, diseases, traffic accidents, and natural disasters [1]. At present, the research of the medical rehabilitation industry focuses on the design and development of intelligent prosthesis and its structure optimization [2,3].

Lower limb prosthesis is a complex human–machine co-drive system, which requires that the binding force at the human–machine contact position should not be too strong. Otherwise, it will affect the effect of rehabilitation and even damage the stump [4]. If the movement of prosthesis is not in accordance with the intention of the amputee, the man–machine movement is uncoordinated, and the amputee will contract his muscles consciously to maintain the desired movement. The EMG generated when contracting muscles is directly related to the body’s desired movements, containing rich neuromuscular information [5,6,7].

EMG can reflect the motion intention of the human body, and it is widely used in rehabilitation training of lower limb disabled persons [8,9,10,11]. In order to accurately determine whether the trajectory of EMG-controlled prosthesis is the same as the expected trajectory of the human body, it is very important to determine the acquisition position of the EMG signal of the residual limb [12,13,14,15,16,17].

Most current studies are based on statistical analysis of major muscle groups [18,19,20,21,22], comparing the correlation between EMG of each muscle group, and determining the EMG position which is most relevant to the motion intention. Reference [18] selected four thigh muscles as the EMG source of lower extremity through a large number of repeated experiments. Reference [19] proposed a method for selecting the location of EMG signal based on ANOVA (analysis of variance) and BP (back propagation) neural network. Four surface myoelectric electrodes were applied to collect the EMG of the subject during specific movements, and the optimal EMG measurement location was selected from the subset with the highest motion recognition accuracy and the minimum electrodes. Reference [20] collected sixteen muscles of the upper limbs to recognize the movement intention. However, because of the large amount of information, the difficulty of signal processing increased, and there was a lot of useless information, which affected the speed and effect of recognition. Reference [21] reduced sixteen EMG signal channels to three by using a variance-based method through a large number of experiments. Reference [22] reduced the EMG signal acquisition channel from fifty-seven to five of the upper limbs through the feature extraction and classification of large amounts of data. In the above studies, the EMG acquisition location covers most of the limb’s muscle groups with good research results. As the number of muscle groups increases, the workload of statistical analysis and experimentation will increase dramatically. It is not practical to compare EMG on all muscle positions. On the other hand, as the muscles are destroyed, there is a marked difference between the EMG of a healthy limb and that of a stump [23,24]. The location selection method of EMG collection based on anatomy is not suitable for residual limb. Therefore, it is necessary to select the position of EMG signal acquisition of residual limb muscle by an appropriate method.

The movement of the stump is the result of multi-muscular coordination [25]. Each muscle group can act independently and be interconnected to form a complex network system with a similar organization and complex intermediate connections. In recent years, the theory of complex networks [26,27,28,29] has developed rapidly. Its local and global characteristics can clearly describe the relationship between different elements and the process of information flow. For example, the conversion of EEG (electroencephalogram) to brain network has seen rapid advances in electroencephalography in recent years [30,31]. Therefore, the complex network theory can be applied to analyze the residual limb muscle groups. A complex network is constructed using the EMG of the stump’s muscle group and applied to analyze the spatial distribution and internal relation of the EMG from a global perspective.

**Figure 1:**
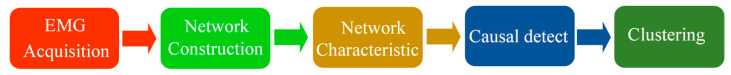
Flowchart of all stages.

By analyzing the EMG of the stump in three motion conditions (appropriate angle, excessive angle, and angle too small), the connection relationship between different EMG channels is explored by the mutual information method. The complex relationship between each EMG channel is abstracted into a network, and the relationship between the motion conditions and the nodes of the network is discussed by analyzing the network topology and evaluating the importance of nodes. In order to analyze the electrophysiological spatial distribution and working mechanism of muscle coordination during limb movement, the Convergence Cross-Mapping (CCM) algorithm is used to analyze the cause and effect of important nodes to construct a directional network of the residual limb. The technical route of this article is shown in Figure 1.

## 2. Materials and Methods

### 2.1. EMG Acquisition

**Figure 2:**
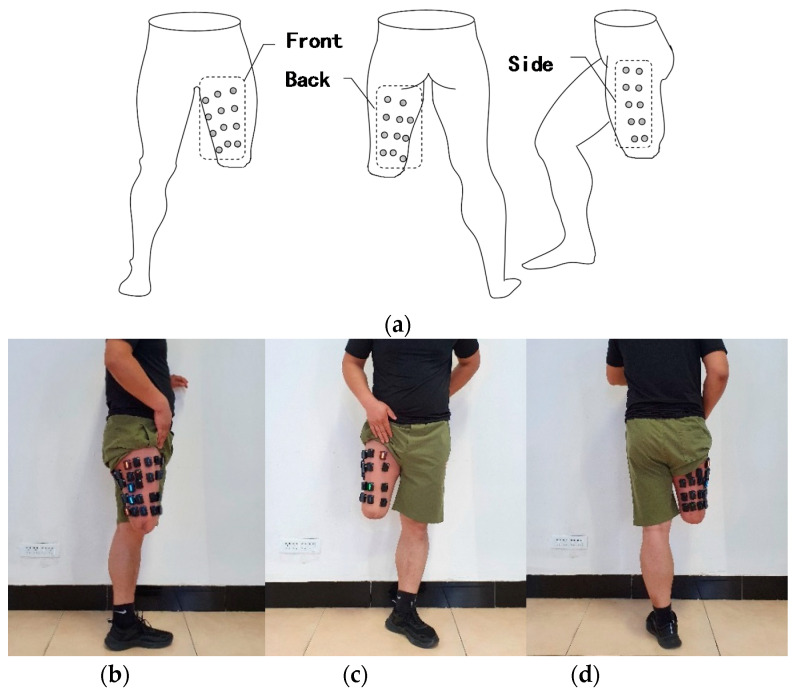
EMG electrodes distribution of the residual limb, (**a**) schematic diagram of electrode distribution, (**b**) side view, (**c**) front view, (**d**) back view.

As shown in Figure 2, through the size of the surface area of the residual limb and in order not to interfere with the signals between the two acquisition modules, 33 EMG electrodes, which are denoted as V1~V33, are placed in the residual limb (Wireless acquisition system of Delsys in USA, acquisition frequency 2000 Hz). Starting from the front of the residual limb, EMG electrodes V1~V12 are placed, EMG electrodes V13~V23 are placed on the back of the residual limb, and EMG electrodes V24~V33 are placed on the outer side of the residual limb. Define each electrode as a network node, and the number of node is n. The EMG signal of each node is collected, and a network is constructed according to the nodes’ correlation characteristics.

Several subjects completed the experiment. All subjects gave their informed consent for inclusion before they participated in the study. The study was conducted in accordance with the Declaration of Helsinki (HEBUThM BC2020003). In order to collect the high-density EMG information of the stump surface and avoid the influence of the prosthesis on the movement, the subjects did not wear the prosthesis during this experiment.

**Figure 3:**
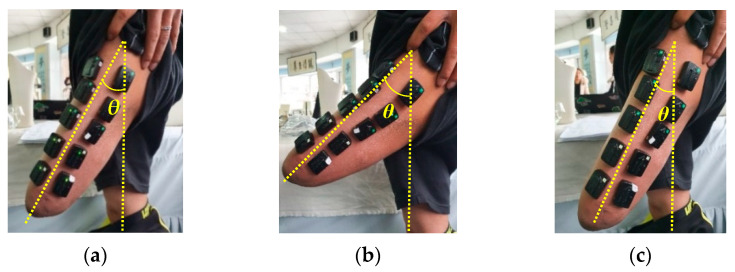
The three movement conditions. (**a**) appropriate angle, *θ* = 35°, (**b**) excessive angle, *θ* = 45°, (**c**) angle too small, *θ* = 25°.

Three motion conditions are defined, as shown in Figure 3, which simulate three kinds of human–prosthesis disharmony. When the subjects wear the prosthesis to move normally, the appropriate angle is measured to be 35 degrees. Then, the angle at which the subjects step when applying traction and resistance is measured. Take the average value of the angle, we get three movement modes:

(1) Appropriate angle: When the residual limb swings naturally, the expected angle is about 35°.
(2) Excessive angle: The movement angle of the stump is greater than the expected angle. A forward traction force is exerted during the swing of the stump, and the swing angle is about 45°.
(3) Angle too small: The movement angle of the stump is less than the expected angle. A backward resistance is applied during the swing of the stump, and the swing angle is about 25°.

**Figure 4:**
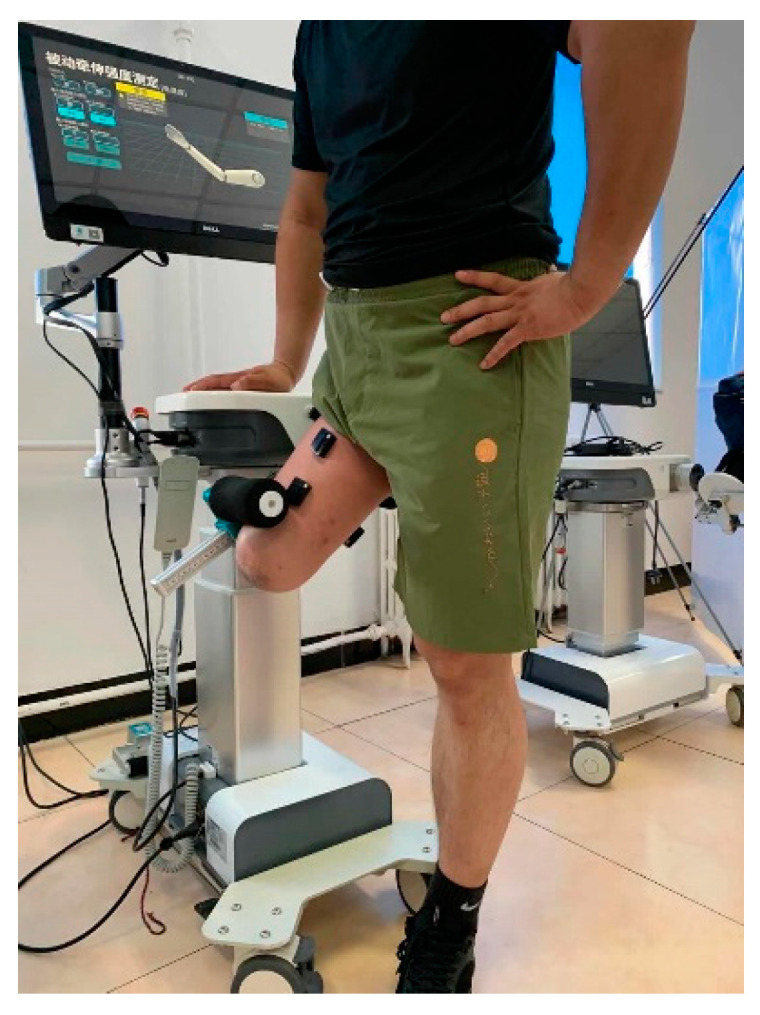
Simulated exercise experiment of the residual limb by the rehabilitation robot.

As shown in Figure 4, the knee joint rehabilitation robot of EXTREME MEDICAL TECH Company (Hangzhou, China) was applied to help the subjects simulate the above three movement conditions. The subjects were asked to perform the leg swing with 35°. (1) Appropriate angle: the angle of robot is set at 35°. The subject follows the rehabilitation robot to complete the leg swing. (2) Excessive angle: the angle of robot is set at 45°. The rehabilitation robot provides a forward traction to the stump of the subject after the leg swing with 35°, and the stump is forced up to 45°. (3) Angle too small: the angle is set at 25°. The rehabilitation robot provides a back resistance to the stump of subject to limit its leg-swinging motion to 25°. Each movement is divided into three stages: lifting (1.5 s), maintaining (1 s), and putting down (1.5 s). These three movements are repeated 10 times at the same speed, and the subject rests 60 s after every five exercises to avoid the influence of muscle fatigue on the experiment. The Butterworth filtering was performed on the collected EMG signal to eliminate noise and remove zero drift.

### 2.2. Functional Network Construction

The key to abstract the residual muscle group system into a network is to clarify the connection relationship between the nodes and analyze the correlation between the two channels by using mutual information [32]. Two EMG channels S and Q are defined. The EMG data of channel S is $s_{1}$, $s_{2}$ …, $s_{n}$, and the probability distribution is $P_{s}\left(s_{1}\right)$, $P_{s}\left(s_{2}\right)$, …, $P_{s}\left(s_{n}\right)$. The EMG data of channel Q is $q_{1}$, $q_{2}$, …, $q_{n}$, and the probability distribution is $P_{q}\left(q_{1}\right)$, $P_{q}\left(q_{2}\right)$, …, $P_{q}\left(q_{n}\right)$. The joint distribution probability $P_{sq}\left(s,q\right)$ is calculated.

According to Shannon’s information theory [33], the entropy analysis can solve information quantification. The information entropy of S and Q is defined as:

(1) $$H\left(S\right)\;=\;-\sum_{i}P_{s}\left(s_{i}\right){log}_{2}P_{s}\left(s_{i}\right){}^{\circ}$$

(2) $$H\left(Q\right)\;=\;-\underset{j}{\sum }P_{q}\left(q_{j}\right){log}_{2}P_{q}\left(q_{j}\right)$$

The information entropy reflects the amount of information contained in each EMG channel, and the joint entropy of channel S and channel Q is calculated as:

(3) $$H\left(S,Q\right)\;=\;-\underset{i,j}{\sum }P_{sq}\left(s_{i},q_{j}\right){log}_{2}P_{sq}\left(s_{i},q_{j}\right)$$

The joint entropy reflects the joint probability distribution of the two channels. The mutual information (*MI*) between the two channels is calculated as:

(4) MI(S,Q) = H(S)+H(Q)−H(S,Q)

when MI = 0, and the EMG of two channels are independent. The larger MI, the stronger the correlation between the EMG of two channels. The n×n adjacency matrix $A\left(a_{ij}\right)$ is constructed according to the correlation characteristics between the signals. The rows and columns represent nodes, and $a_{ij}$ represents *MI* between channel i and channel j. The $a_{ij}$ is normalized, and the complex network formed by the matrix $A\left(a_{ij}\right)$ is simplified into a sparse binary, unweighted, undirected form by selecting an appropriate threshold, TH. If the mutual information (MI) is greater than TH, then $a_{ij}\;=\;1$, and a connection edge is established between channel i and channel j. Otherwise, $a_{ij}\;=\;0$, and no connection edge is established.

The threshold (*TH*) selection is critical and directly affects the statistical characteristics and topology of the muscle function network. The selection of *TH* follows the following principles:

(1) Ensure network connectivity and avoid too many isolated points.
(2) Network average degree <k> (Equation (6)) is greater than 2 lnn.

### 2.3. Network Characteristics

The analysis of the nodal characteristics [34] of muscle functional networks helps to understand the differences between networks in different movements and plays an important role in determining the connections between key muscle nodes.

(1) Node degree

Node degree, $k_{i},$ represents the number of edges associated with node *i*.

(5) $$k_{i}\;=\;\overset{n}{\underset{j\;=\;1}{\sum }}a_{ij}$$

The greater the node degree, the more important the node in the network is.

Calculate the average of the degrees of all nodes and record it as the average degree of the entire network as:

(6) $$\langle k\rangle \;=\;\frac{1}{n}\overset{n}{\underset{i\;=\;1}{\sum }}k_{i}$$

In a network with *n* nodes, the proportion P(k) of nodes with degree *k* is:

(7) $$P\left(k\right)\;=\;\frac{n_{k}}{n}$$

(2) Clustering coefficient

Clustering coefficient, $C_{i},$ is applied to describe the degree of network aggregation. Take the average of all $C_{i}$ in the network to get the network aggregation coefficient C:

(8) $$\;C_{i}\;=\;\frac{2E_{i}}{k_{i}\left(k_{i}-1\right)}$$

(9) $$C\;=\;\frac{1}{n}\overset{n}{\underset{i\;=\;1}{\sum }}C_{i}$$

where $E_{i}$ is the proportion of connections between the neighbors of node i, and $k_{i}\left(k_{i}-1\right)/2$ is the maximum of $E_{i}$ for undirected networks.

(3) Average path length

Average path length, L, represents the average distance between any two nodes:

(10) $$L\;=\;\frac{1}{n\left(n-1\right)}\underset{i\neq j}{\sum }d_{ij}$$

where $d_{ij}$ represents the number of edges on the shortest path connecting node i and node j, that is, the distance between two nodes.

### 2.4. Node Contraction Method

Define the cohesion of network M as:

(11) $$\partial \left(M\right)\;=\;\frac{1}{n\times L}\;=\;\frac{n-1}{\sum_{i\neq j}d_{ij}}$$

Calculate the average path length between all pairs of nodes and the initial cohesion of the network. Merge node i and node-set V directly connected to node i into one node. All edges connected to set V are directly connected to the new node, then, calculate the importance of node i [35]:

(12) $$IMC\left(i\right)\;=\;1-\frac{\partial \left(M\right)}{\partial \left(M\times i\right)}\;=\;1-\frac{\frac{1}{n\times L\left(M\right)}}{\frac{1}{\left(n-k_{i}\right)\times L\left(M\times i\right)}}$$

where M×i is the new network after node i contracts in network M. The higher the contraction’s importance, the higher the degree of network cohesion after the contraction. Therefore, it can be used to evaluate the importance of the node.

### 2.5. Convergent Cross-Mapping Algorithm

The CCM algorithm can clearly express the relationship between variables in a nonlinear coupled system and is suitable for detecting EMG signals’ causality. According to the time delay embedding theorem, in the same dynamic system, the two related variables, x and y, and the corresponding reconstructed manifolds, $M_{x}$ and $M_{y}$, are all differential isomorphic to the manifold, M, of the original system. Since both $M_{x}$ and $M_{y}$ are differential isomorphic to M, for each element $x\left[t_{1}\right]$, $x\left[t_{2}\right]$, …, $x\left[t_{i}\right]$ in $M_{x}$, there are also $y\left[t_{1}\right]$, $y\left[t_{2}\right]$, …, $y\left[t_{i}\right]$ corresponding to each element in $M_{y}$. At some point on $M_{x}$, its corresponding nearest neighbor cannot be accurately mapped to the nearest neighbor of the corresponding point on $M_{y}$, and the distance is very far, so x contains very little information about y. On the contrary, the nearest neighbor corresponding to a point on $M_{y}$ can be accurately mapped to the nearest neighbor of the corresponding point on $M_{x}$, then the information of x is contained in y, and a one-way causal relationship from x to y is formed.

Suppose there is a d-dimensional (d≤N) manifold, M, that changes with time in the N-dimensional space. {x} is the sequence of length, L, produced by the projection of the manifold, M, on a certain dimension, and {y} is the sequence of the same length produced by the projection of the manifold, M, on another dimension. To reconstruct these two sequences, set the reconstruction dimension as E and the delay time as τ. The coordinates of the reconstructed sequence at time t can be obtained as:

(13) x(t) = 〈x[t],x[t−τ],⋯,x[t−(E−1)τ]〉

(14) y(t) = 〈y[t],y[t−τ],⋯,y[t−(E−1)τ]〉

From Equations (13) and (14), the reconstructed manifolds $M_{x}\;=\;\left\{x\left(t\right)\right\}$ and $M_{y}\;=\;\left\{y\left(t\right)\right\}$ can be obtained. From the Takens embedding theorem, the manifolds $M_{x}$, $M_{y}$, and M are differential homeomorphisms. According to the simplex projection operator theory [36,37], $\overset{\wedge }{x}\left[t\right]|M_{y}$ is defined as the cross-mapping estimation of y[t] through Equations (14)–(16) to x{t}. Find the E+1 points closest to y(t) from the manifold $M_{y}$, which corresponds to $x\left[t_{i}\right]$ on x[t]:

(15) $$\overset{\wedge }{x}\left[t\right]|M_{y}\;=\;\overset{E+1}{\underset{i\;=\;1}{\sum }}w_{i}x\left[t_{i}\right]$$

(16) $$w_{i}\;=\;\frac{m_{i}}{\sum_{j\;=\;1}^{E+1}m_{j}}$$

(17) $$m_{i}\;=\;exp\{\frac{-d[y\left(t\right),y\left(t_{i}\right)]}{d[y\left(t\right),y\left(t_{i}\right)]}\}$$

where $d[y\left(t\right),y\left(t_{i}\right)]$ represents the Euclidean distance between y(t) and $y\left(t_{i}\right)$ on the manifold. This allows for calculation of the correlation coefficient between $\overset{\wedge }{x}\left[t\right]|M_{y}$ and x[t], with the following formula:

(18) $$r\;=\;\frac{\sum_{i\;=\;1}^{L}\left(x\left[i\right]-\bar{x\left[i\right]}\right)\left(\hat{x}\left[t\right]|M_{y}-\bar{\hat{x}\left[t\right]|M_{y}}\right)}{\sqrt{\sum_{i\;=\;1}^{L}{\left(x\left[i\right]-\bar{x\left[i\right]}\right)}^{2}\sum_{i\;=\;1}^{L}{\left(\hat{x}\left[t\right]|M_{y}-\bar{\hat{x}\left[t\right]|M_{y}}\right)}^{2}}}$$

with the increase of the time series length, L, $\overset{\wedge }{x}\left[t\right]|M_{y}$ gradually converges to x[t], that is, the correlation coefficient, r, converges to a number greater than 0 and less than 1. There is a causal relationship from x to y. When r is close to one, then the causality is large.

## 3. Results

### 3.1. Network Establishment and Characteristics Analysis

**Figure 5:**
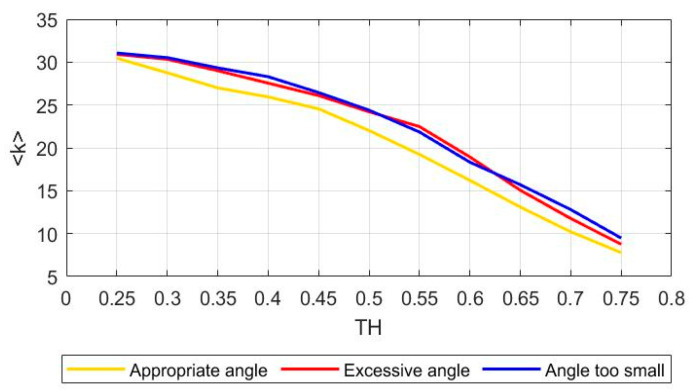
Changes in average node degree under different thresholds.

**Figure 6:**
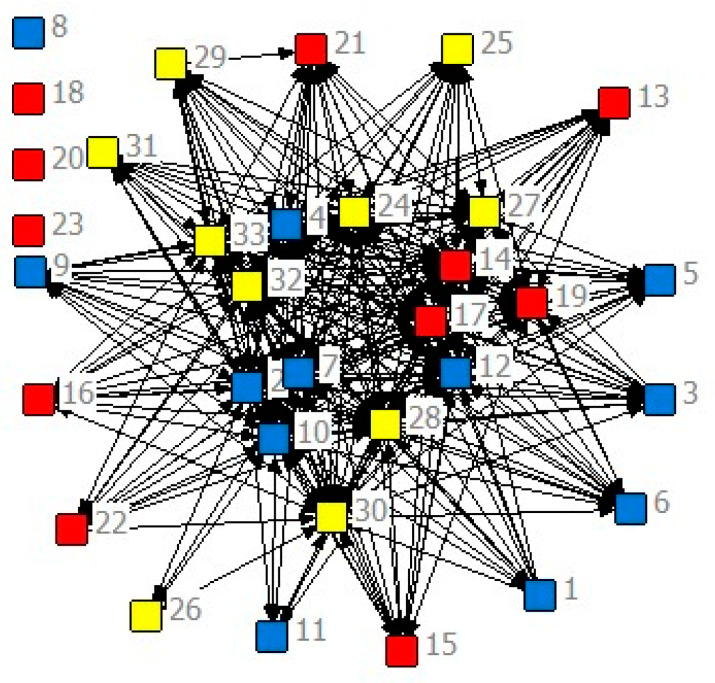
The network of appropriate angle with the threshold *TH* = 0.60. There are four isolated nodes, V8, V18, V20, and V23.

Because the impact of the prosthetic limb on the stump occurs mainly in the lifting phase, the EMG signals of the lifting phase are analyzed. The EMG signals are recorded by 33 EMG electrodes, the entropy of each channel is calculated, the mutual information between any two channels is analyzed, and the weighted adjacency matrix of the muscle function network is obtained. By analyzing the main characteristic in the process of threshold, TH, from 0 to 1, the appropriate threshold (TH = 0.55) is selected. Threshold, *TH,* needs to meet the following conditions: (1) <k> needs to be greater than 2 lnn = 6.99 (Figure 5). (2) Ensure network connectivity and avoid too many isolated points. When the threshold, *TH,* exceeds 0.60, isolated points begin to appear in the network (Figure 6). When the threshold, *TH,* is 0.55, the three movements have the biggest difference (*p* < 0.0001).

**Figure 7:**
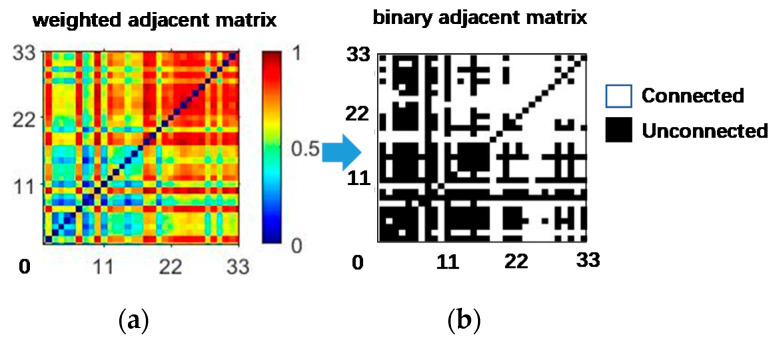
The adjacency matrix of the muscle function network. The weighted adjacent matrix becomes a binary adjacent matrix after proper threshold processing (*TH* = 0.55). (**a**) weighted adjacent matrix, (**b**) binary adjacent matrix.

The mutual information matrix is converted into a binary matrix (Figure 7). The black area indicates that the mutual information value is below the threshold (there is no connectivity between corresponding nodes). The white area means that the mutual information value is higher than the threshold (the network’s corresponding nodes are connected).

**Figure 8:**
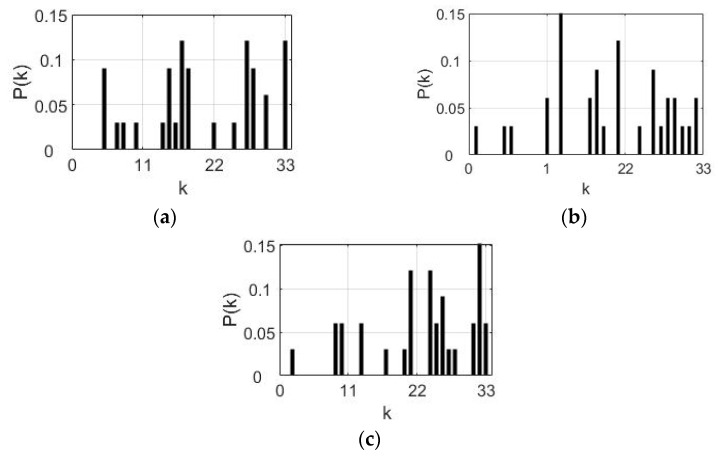
The degree distribution in the three kinds of movements: (**a**) appropriate angle, (**b**) excessive angle, (**c**) angle too small.

Calculate the node degree distribution P(k) of the network in three kinds of motion (appropriate angle, excessive angle, angle too small). As shown in Figure 8, k represents the degree value, and P(k) represents the distribution probability of each degree. In the movement condition of appropriate angle, the node distribution is relatively uniform, and the connections between nodes are partly strong and partly weak (Figure 8a). In the movement condition of excessive angle, the nodes are mainly distributed in the region with middle and high node degree, and only a few nodes have small node degrees, which indicates that the connection of the network is tight (Figure 8b). In the movement condition of angle too small, the node moves to the area with the higher node degree compared to Figure 8b, the value of the overall distribution is high, and the connection between nodes is the tightest (Figure 8c). The results show that when the residual limb is not disturbed by external forces, the residual limb’s movement is the most steady. When the residual limb is subjected to external forces, whether it is forward traction or backward resistance, the residual limb muscles respond to external changes. The software SPSS 25.0 is used to analyze the variance of the average degree characteristics of the three networks of all subjects. The results show that there is a significant difference between the appropriate angle and the other two movements (*p* < 0.04).

**Figure 9:**
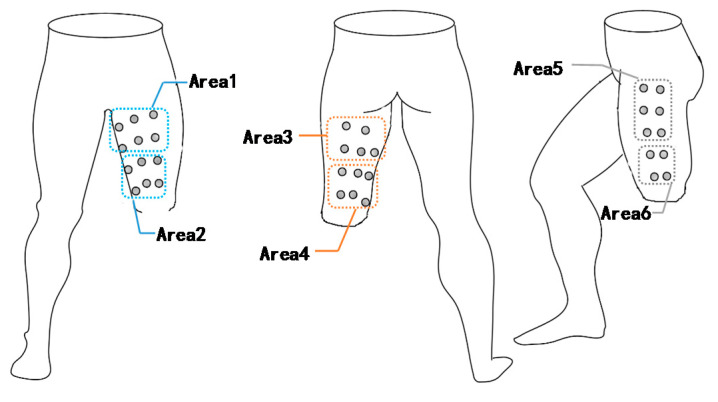
The surface of the residual limb is divided into six areas. The standard for distinguishing the areas is the distance from the wound. Area 1 and Area 2 are on the front, Area 3 and Area 4 are on the back, and Area 5 and Area 6 are on the outer side.

**Figure 10:**
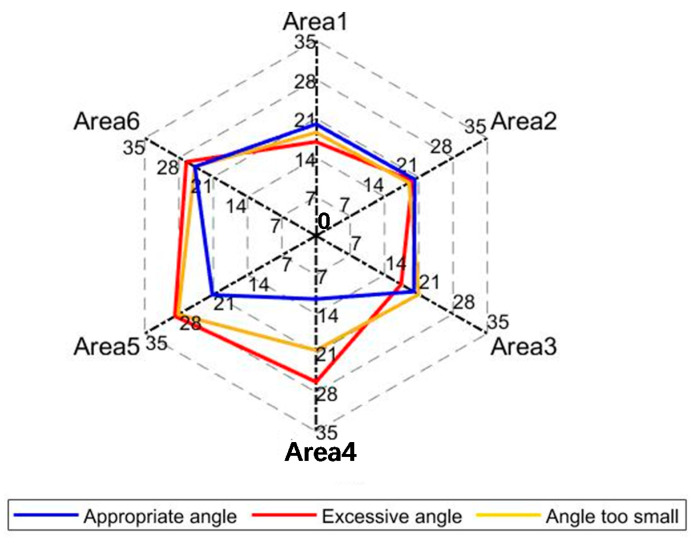
The average degree of the six areas is analyzed. The value change in the radar chart from inside to outside indicates the change of average degree value. The smaller value is near the center, and the greater value is near the outside. Six corners represent six areas. The blue line represents the appropriate angle, the red line represents the excessive angle, and the yellow line represents the angle too small.

To study whether the muscles close to the wound or far away from the wound are different during exercise, the residual limb’s three surfaces are divided into six areas (Figure 9). These six areas are divided due to distance from the wound and the number of nodes. Ensure that the number of nodes in each area is roughly the same. The average degree <k> of the different areas in the three types of exercise is analyzed, as shown in Figure 10.

As shown in Figure 10, there are differences in the average node degree in different areas. There is little difference between Area 1 and Area 2, indicating that the frontal muscles are not significantly affected by trauma.

Area 3 and Area 4 are different. When doing different exercises, <k> is also different. When the angle is appropriate, the value of <k> is obviously lower than the average value, so the back area is greatly affected by limb amputation.

There are also differences in the two areas on the outer side (Area 5 and Area 6). Whether it is close to the wound or far away from the wound, the mean value of <k> is greater than the overall mean value, and the <k> far away from the wound is larger. From the above results, when the residual limb is amputated, the muscles far away from the wound act obviously higher than those close to the wound. Moreover, the side muscles play the most critical role in the residual limb’s movement, which is consistent with the actual situation.

### 3.2. Node Importance Evaluation

The node importance represents the criticality of the node in the network. The higher the importance of the node, the higher the contribution of the node in the network, and the node has the strongest influence on the network. Evaluate the importance of nodes in the network and discover important nodes in the network. The key nodes of information perception are related to their central location and the time when information is exchanged with other nodes.

**Figure 11:**
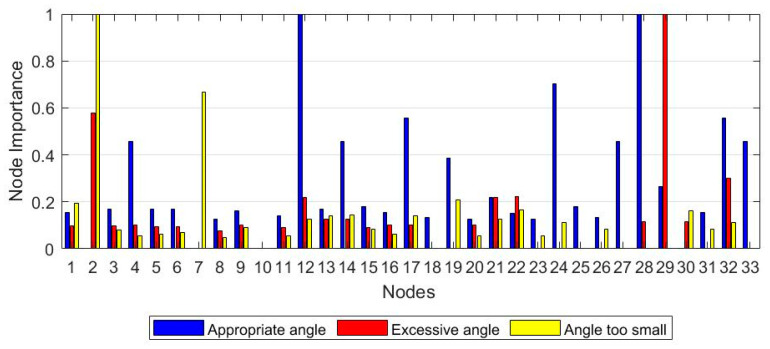
The importance of 33 nodes. The blue bar represents the appropriate angle, the red bar represents the excessive angle, and the yellow bar represents the angle too small.

The node contraction method is used to analyze the node importance under three movement conditions (appropriate angle, excessive angle, and angle too small). The node importance of each movement is calculated by Equation (12), and the result is shown in Figure 11. The higher the node’s importance, the more critical the node’s position, and the more significant the contribution to the entire network. Therefore, a threshold can be set, and nodes with greater importance than the threshold can be selected to construct a network.

**Figure 12:**
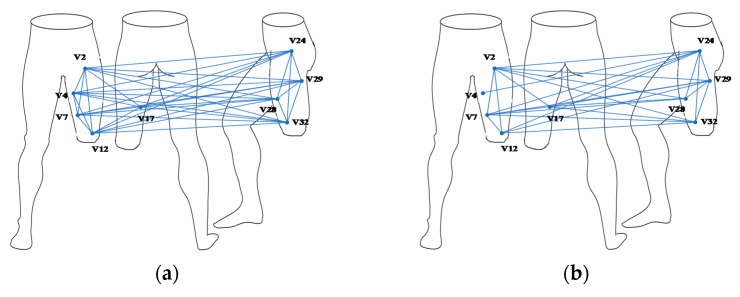

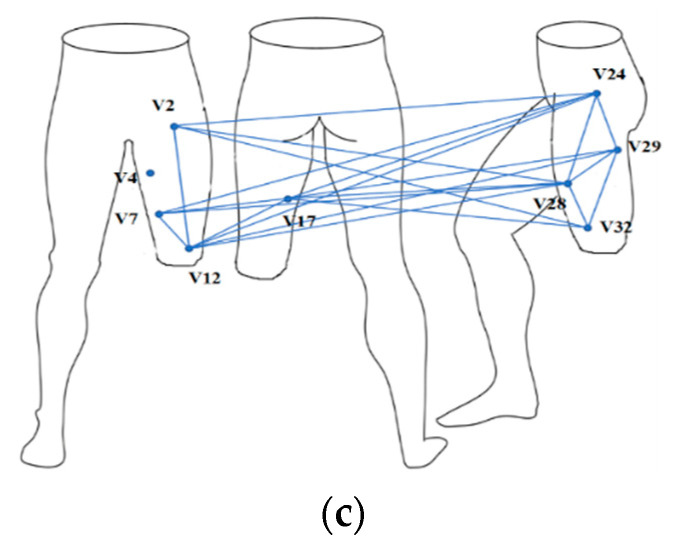
Muscle functional network of selected nodes: (**a**) appropriate angle, (**b**) excessive angle, (**c**) angle too small.

The nodes whose values are greater than the threshold value of 0.5 are selected based on the network size and node importance distribution. Nine nodes with significant differences (V2, V4, V7, V12, V17, V24, V28, V29, and V32) are chosen to construct a muscle function network for each of the movement conditions, as shown in Figure 12. When the angle is appropriate, the connection between important nodes is the tightest and smoothest (Figure 12a). In excessive angle and the angle too small, the connection between the nodes will react due to external forces’ appearance. When the angle is too small, the connection between nodes is the weakest, which is consistent with the results of the previous study on the degree distribution (Figure 12c). Each movement state has apparent differences, which is helpful for further analysis and application of node information.

As shown in Figure 12, the nodes are mainly distributed on the residual limb’s front and side muscles, and the back muscles are the least distributed. The connection degree of the nodes at the frontal muscles is not related to whether they are close to the wound. Most of the key nodes at the side muscles are located far away from the wound and are most connected with other nodes. The back muscle has only one key node which is located away from the wound. The above results are consistent with the results of node degree differences in different areas. As shown in Figure 12, the network connections of the three movements are different. In the appropriate angle, the connection between nodes is the closest. In the excessive angle, the degree of connection is weakened. When the angle is too small, the connection is the weakest. The above results are the same as the results of the network characteristic analysis in the previous section.

(1) Appropriate angle: the residual limb performs a regular leg swing exercise, the muscle contraction is the strongest, all the muscles work together, and the movement is the most stable.
(2) Excessive angle: the side muscles act slightly more potent than the front when the residual limb receives forward traction.
(3) Angle too small: the residual limb is subjected to backward resistance. To reduce the discomfort to not cause damage to itself, the muscle contraction is not apparent.

### 3.3. Construction of Directed Network

**Figure 13:**
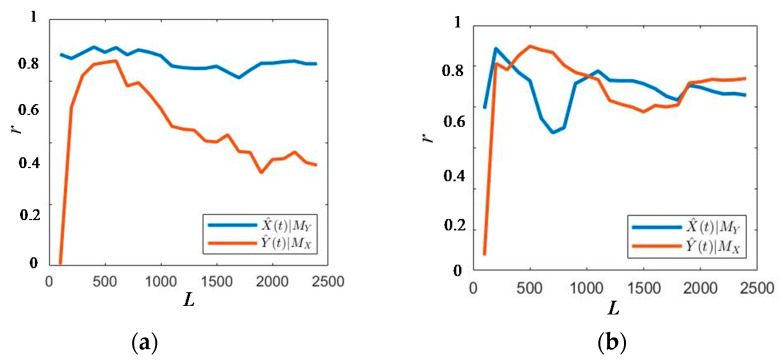

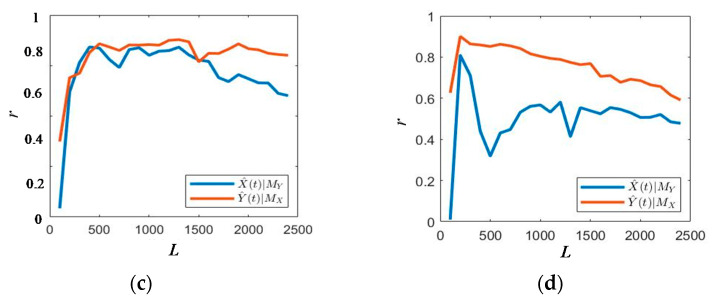
Convergent cross-mapping situation. (**a**) *Mx*–*My* > 0.3, (**b**) *Mx–My* < 0.1, (**c**) 0.1 < *Mx–My* < 0.3, *Mx* > 0.5, and (**d**) 0.1 < *Mx–My* < 0.3, *Mx* < 0.5.

The two sets of EMG signals, x and y, mentioned in Section 2.5, can be regarded as variables related to one system, so as the length of the data increases, the correlation coefficient, *r* (Equation (18)), will converge to a value greater than 0 and less than 1. When the correlation coefficient, *r,* is closer to 1, and the greater the coefficient differential value between the two detected channel EMG signals, the stronger the causal relationship. As shown in Figure 13, there are four possible convergence cross-mapping situations between the EMG signals in the residual limb movement experiment.

The causality is defined based on the differential value:

(1) When *Mx–My* > 0.3, the strength of causality from *x* to *y* is much stronger than the strength of causality from *y* to *x*, so there is a one-way causal relationship from *x* to *y*.
(2) When *Mx–My* < 0.1, there is not only the information containing *y* in *x* but also the information containing *x* in *y*, and the causal relationship strength is similar between the two, so there is no one-way causal relationship between *x* and *y*, and there is no information flow.
(3) When 0.1 < *Mx–My* < 0.3, *Mx* > 0.5, there is no one-way causal relationship.
(4) When 0.1 < *Mx–My* < 0.3, *Mx* < 0.5, there is a one-way causal relationship from *x* to *y*.

**Figure 14:**
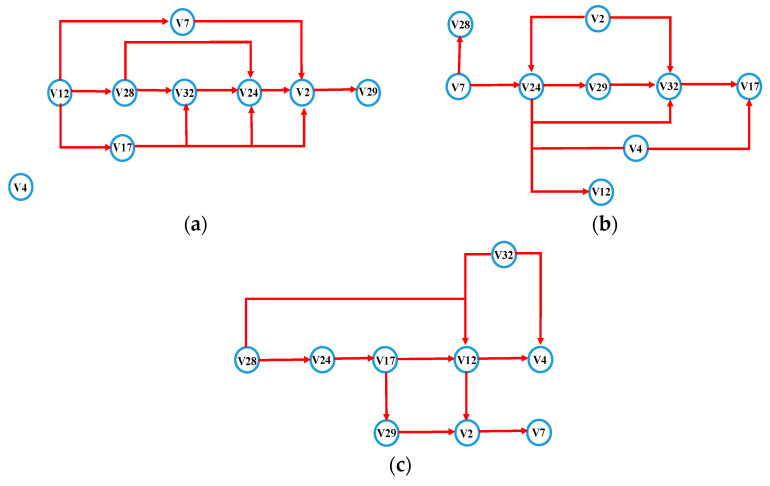
Information flow diagram of the three movement conditions: (**a**) appropriate angle, (**b**) excessive angle, (**c**) angle too small.

By causality detection, if there is a causal relationship between X and Y, X is the cause, and Y is the effect. There is information flowing from X to Y, and due to the loss in the information flow process, X contains more information than Y. According to the convergent cross-mapping evaluation results, the information flow network of the three movements is constructed, as shown in Figure 14.

(1) Appropriate angle: the arrow pointing represents the direction of information flow, the starting end of the arrow is the cause, and the pointing end is the effect. V12 is the top of the information outflow point, which contains the most information. In addition to V12, the points V7, V17, and V28 are also at the front end of the information flow, indicating that these nodes also contain relatively more motion information. The information inflow points V2 and V29 are located at the bottom of the information flow. There are more information inflows, indicating that participation is of secondary importance when the angle is appropriate.
(2) Excessive angle: V7 is the vertex of the information outflow point. V2, V24, and V28 also have more information outflow, indicating that these nodes contain the most motion information when the angle is too large.
(3) Angle too small: V28 is located at the starting point of information flow and is relatively most important in the movement process. V28, V24, and V32 nodes contain more motion information when the angle is too small. V4 and V7 are at the end of the information flow chain when the angle is too small.

**Figure 15:**
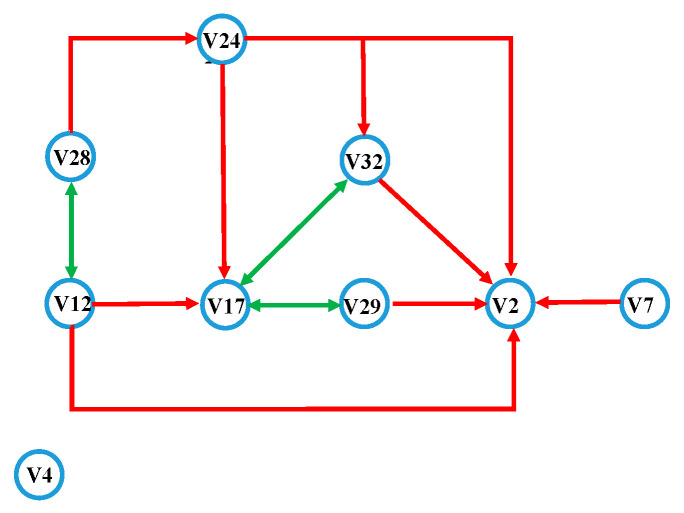
A directed network on the surface of the residual limb. The red line represents the unidirectional flow line, and the green line represents the bidirectional flow line.

Synthesizing the information flow diagrams, a directed network of the stump in the three movements (appropriate angle, excessive angle, and angle too small) can be constructed, as shown in Figure 15.

The topmost node of the network contains the most information, so it is most critical. As shown in Figure 15, V12 and V28, as the topmost layer of the directed network, are the nodes with the fastest reach of motor nerve information transmission, and they contain the most information. V17 and V24 are on the second layer of the directed network, and they only contain less information than V12 and V28. Because there is an information flow from V24 to V17, V24 contains more information than V17. V2 is located at the bottom of the network, which means it contains the least information. Because V29 and V32 only have one layer of connection with V2 and there are two layers between V17 and V2, the strength of V29 and V32 are equivalent and both weaker than V17. V7 has one layer of connection with V2, but there is no network layer before V7, so V7 is more important than V29 and V32. V4 does not participate in the construction of the directed network, and it is located at the end of the ranging. It can be determined that the importance of the residual limb muscles in the movement of thigh amputation patients is: V12 = V28 > V24 > V17 > V7 > V32 = V29 > V2 > V4.

**Figure 16:**
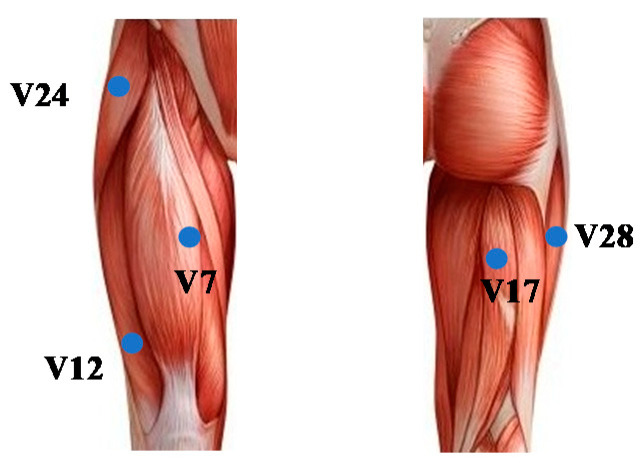
Key nodes’ distribution.

Too many acquisition modules inside the prosthesis will result in a complex structure of the prosthesis. In order to optimize the structure of the prosthesis and collect enough information, the front, back, and side of the residual limb need to have collection modules. The three conditions of the number of modules, module distribution area, and directed network are taken into consideration. There are the following schemes for node selection: The information outflow point is the node where the movement information reaches the fastest, and the outflow point contains more movement information and is more critical in the movement process. According to the information outflow point and information inflow point of the directed network, the five nodes V12, V28, V24, V17, and V7 are selected as the residual limbs’ key nodes. The key nodes are mainly distributed in the rectus femoris, vastus lateralis, tensor fascia lata, and semitendinosus, as shown in Figure 16.

### 3.4. Analysis of Movement Difference

To quantify the differences in every movement, the muscle function network of key nodes is analyzed. The clustering coefficient, C, the average degree, <k>, and the average path length, L, of the three movement networks are calculated. The results are shown in Table 1.

The higher the clustering coefficient, the better the connectivity of the network. The larger the average degree, the stronger the correlation between the channels, and the more pronounced the promoting effect of the network topology on cooperative behavior. The average path length represents the average value of the distance between any two nodes. The shorter the length, the faster the information flows. It can be seen from Table 1 that there are significant differences in the muscle function network characteristics of the key nodes of each movement. When the angle is appropriate, the correlation between nodes is the highest, which is higher than when the angle is too large and when the angle is too small. It shows that the muscles cooperate smoothly without being affected by the excessive restraint of the prosthesis. When the angle is too large and the angle is too small, the clustering coefficient and the average path length are significantly higher than when the angle is appropriate (*p* < 0.001), indicating that the connectivity between nodes is enhanced when affected by external forces. The transmission rate of information in the muscle function network is increased to deal with the outside external force influence.

In summary, when no external force is applied, the correlation between the residual limb muscles is stronger, and the coordination between the muscles is more coordinated and the movement is steadier. When affected by the prosthesis’ excessive restraint, the connectivity between the residual limb muscles is better, and the information flow between the muscles is more efficient to respond to the external force interference and make corresponding stress responses.

**Figure 17:**
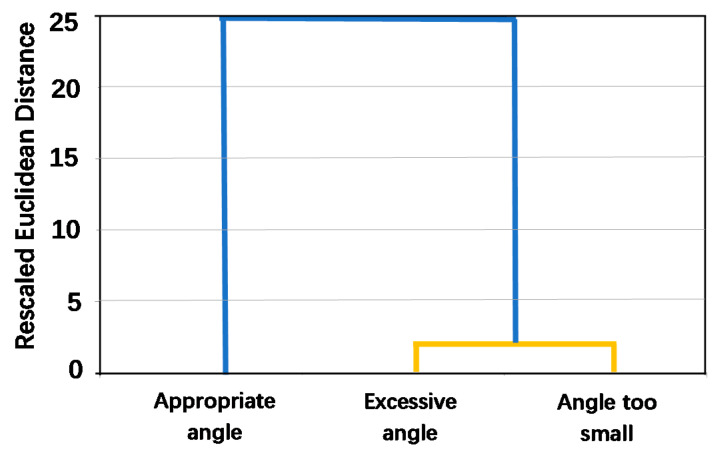
Dendrograms of the clustering for different movements. The two ends of the yellow line are classified into one category. The end of the blue line is classified into one category.

By using the classification function of the software SPSS 25.0, the three movements are clustered using the network characteristics of Table 1, and the clustering results are shown in Figure 17. The abscissa represents the type of movement variable. The ordinate represents the rescaled Euclidean distance. Take the distance as 2, it can be divided into two categories, and the clustering results are: {appropriate angle}, {excessive angle, angle too small}. The network characteristics can be used to divide the limb’s movement into two categories: affected by the excessive restraint of the prosthesis and not affected by excessive restraint. The results are consistent with the above research results.

## 4. Discussion

The control trajectory of the intelligent lower limb prosthesis may be different from the expected trajectory of the prosthesis wearers. In general, the wearers need to adjust themselves to fit the artificial limb, which may cause fatigue and even safety problems for amputees. In this study, the gait of the residual limb after being subjected to the excessively strong restraint of the prosthesis was divided into three types: appropriate angle, excessive angle, and angle too small. The correlations of multi-channel EMGs were analyzed by the mutual information method, and the complex system of residual muscle group was abstracted into a muscle function network. The features of the muscle function network of three kinds of movements were analyzed, and the importance of nodes was evaluated.

A directed network was constructed after each important node’s causal analysis by using the convergent cross-mapping method. The accurate decoding of the residual limb’s movement information was realized, and the real-time performance and accuracy of the sensing function network were improved. The research results show an absolute difference between the movement subject to the excessively strong restraint of the artificial limb and the movement that is not restrained.

The muscles close to the residual limb wound are different from those far away from the wound. The muscles which play a crucial role are also affected by the amputated wound. This method can quickly and effectively determine the smart prosthesis’ EMG collection location, avoiding the one-sidedness of measuring the importance of nodes by the node degree and bridge coefficient and the high cost of node betweenness evaluation. It can accurately distinguish whether there is an excessive binding force between humans and prosthesis by analyzing the muscle function network characteristics and can avoid secondary damage to the residual limb due to uncoordinated motion and provide state recognition results or triggers for the intelligent prosthetic control command. The establishment and analysis of residual limb EMG function network and directed network offer new ideas and new methods for decoding the relationship between neural control and body movement, and it provides an effective method for simplifying the position of the EMG sensors of the dynamic lower limb prosthesis and optimizing the internal space structure of the prosthesis.

The experiments have been carried out on three subjects only, and hence the results need further validation. The methodology used has, to the best of our knowledge, never been applied on motion coordinated analysis for amputees.

This paper selected the key nodes of the residual limb, and the next step needs to verify the effectiveness of the key nodes. It is planned to use multiple methods such as time domain, frequency domain, and nonlinearity to extract feature values at key nodes, and then apply multiple classifiers for feature recognition.

## Figures and Tables

**Table 1:** Muscle functional network characteristics of key nodes.

Movement	Clustering Coefficient, C	Average Degree, <k>	Average Path Length, L
appropriate angle	0.69 ± 0.029	28.8 ± 2.23	1.32 ± 0.12
excessive angle	0.87 ± 0.057	25.2 ± 3.03	1.22 ± 0.33
angle too small	0.80 ± 0.034	26.2 ± 2.44	1.24 ± 0.23
